# Which youth with suicidal ideation will go on to make a suicide attempt? A systematic review

**DOI:** 10.1016/j.neurot.2026.e01034

**Published:** 2026-08-18

**Authors:** Daniel Dobin, David A. Brent

**Affiliations:** a Center for Suicide Prevention, Johns Hopkins Bloomberg School of Public Health, Baltimore, MD, USA; b Johns Hopkins Krieger School of Arts and Sciences, Baltimore, MD, USA; c University of Pittsburgh School of Medicine, Department of Psychiatry, 3811 O’Hara St BT311, Pittsburgh, PA, 15213, USA

**Keywords:** Suicidal ideation, Behavior, Assessment, Prediction, Prevention

## Abstract

Suicidal ideation is common among adolescents, yet most who experience it never make a suicide attempt. Identifying which youth go on to engage in suicidal behavior remains one of the most pressing challenges in clinical psychiatry. This systematic review synthesizes the evidence on risk and protective factors that are associated with the transition from suicidal ideation to suicidal behavior in adolescents and young adults. We searched five electronic databases from inception through November 2025, yielding 52 studies meeting eligibility criteria. Included studies assessed suicidal ideation at a defined baseline and measured subsequent suicidal behavior, evaluating at least one predictor of that transition in samples with participants under age 30. Across case-control, retrospective, and prospective designs, the strongest predictors of transition were characteristics of the ideation itself, particularly the presence of a plan or intent, high duration and intrusiveness, and lack of controllability. Past suicidal behavior and non-suicidal self-injury were also robust predictors. Psychiatric comorbidity, especially mood disorders combined with externalizing disorders, substance use, or post-traumatic stress disorder, meaningfully increased risk, as did maltreatment, peer victimization, and family conflict. Conversely, parent-child connection, social support, and school connectedness were protective. Findings from this review can inform more precise clinical risk assessment and help identify the highest-priority targets for intervention among youth presenting with suicidal ideation.

## Introduction

The assessment of suicidal risk is one of the most impactful tasks facing mental health professionals. Suicidal ideation is common in adolescents, with one in five youths reporting some level of suicidal ideation (SI) within the past 12 months [1,2]. In a recent international survey of 13,984 college first-year students, the lifetime prevalence of SI was 17.4%, with 75% of suicidal college students having had onset of SI prior to age 16 [3]. In contrast, suicidal behavior, and death by suicide occur much less frequently, which makes prediction challenging.

This paper aims to aid the clinician in the identification of modifiable risk and protective factors to reduce the likelihood of a patient’s transition from SI to suicidal behavior by surveying those aspects of SI and related psychological constructs that are associated with a transition from SI to non-fatal and fatal suicidal behavior. While estimates of the rate of transition from ideation to attempt vary widely, most studies agree that the transitions are most likely to occur within the first year of onset of ideation, and that those transitions are more likely to occur in those patients whose ideation is more frequent, less controllable, and more severe (e.g., development of a plan and method with intent [[4], [5], [6], [7]]). In young adults, the transition from SI to suicidal behavior can take hetergoenous paths, and can take place in period of time of 12 h or less [8,9].

While this paper focuses on the assessment of patients who present with SI, it is important to remember that many patients who die by suicide deny SI at the time of assessment. One meta-analysis showed that slightly under half of suicidal individuals will disclose their ideation [10]. In the Army STARRS study, 67.5% of all soldiers who made suicide attempts denied suicidal ideation at their last assessment prior to their attempt, but their risk profile was otherwise similar to those at high risk for suicidal behavior, namely, at least one diagnosed mental disorder, history of peer victimization within unit, having been responsible for the death of an enemy, and having been in jail while in the service [11]. Therefore, as a caveat to the clinician, the assessment of suicidal risk includes more than SI, but also psychopathology, history of suicidal thoughts and behaviors (STB), current stressors, and other contextual factors.

We review the most commonly occurring risk and protective factors that predict the transition from SI to suicidal behavior. Within each risk category, we examine the evidence from case-control, case-cross-over, and prospective studies that predict the progression from suicidal ideation to suicidal behavior. The risk factors range from distal and proximal stressors (maltreatment, victimization, traumatic stressors, family conflict) to aspects of current and past suicidal ideation, attempt, and non-suicidal self-injury, to psychopathology and substance abuse, psychological traits (e.g., distress tolerance), and constructs relevant to psychological theories of the genesis of suicidal behavior [12]. Finally, we examine the few studies that examine the transition from suicidal ideation to death by suicide. This review aims to help provide the foundation for approaches to assessment, prediction, and prevention of suicidal behavior.

## Methods

### Search strategy

We systematically searched five electronic databases from inception to November 23rd, 2025: PubMed, Embase, APA PsycInfo, Web of Science, and CINAHL. Searches were limited to peer-reviewed studies published in English. The search strategy combined four concept groups using database-specific subject headings and keywords: (1) suicidal ideation, (2) suicidal behavior and suicide attempts, (3) transition/prediction/mechanisms linking ideation to behavior, and (4) adolescents/young adults. A search strategy is available in the Supplementary Material (Table S1). Reference lists of included studies were screened to identify additional eligible articles. Please see Table S2 for a PRISMA guidelines checklist [13]. This systematic review was not registered in PROSPERO because it was conducted as a revision and extension of a prior review.

### Eligibility criteria

Studies were eligible for inclusion if they: (1) assessed clearly defined suicidal ideation (SI) at a distinct time point; (2) reported at least one clearly defined behavioral suicidal outcome (suicidal behavior [SB]), suicide attempt, or related behavioral outcomes) measured after the SI assessment; (3) evaluated at least one mechanism, predictor, or risk factor associated with transition from SI to SB; and (4) examined an adolescent and/or young adult population, with SI and/or SB measured before age 30. We included studies conducted in diverse clinical or community settings, including clinical, community, military, and correctional contexts. Eligible study designs included (but were not limited to) longitudinal, retrospective, prospective, cohort, register-based, case-control, and ecological momentary assessment (EMA) studies.

Studies were excluded if they did not examine an ordered SI to SB framework, did not measure SI distinctly, did not assess a behavioral suicidal outcome after SI, or did not include participants under age 30 at the time SI and/or SB were measured.

### Study Selection

Records were imported into a reference management system, and duplicates were removed prior to screening. Titles and abstracts were screened for eligibility, followed by full-text review of potentially relevant articles.

### Data extraction

Data were extracted using a pre-specified template. Extracted information included study characteristics (e.g., publication year, country, setting), sample characteristics (e.g., age range, sample type), and all reported predictors/mechanisms of transition from SI to SB (including statistical estimates where available). When multiple reports used overlapping samples, the most comprehensive publication was prioritized for extraction, while companion papers were used when they provided unique predictors or outcomes.

### Data synthesis

Given the expected heterogeneity in study designs, measurement approaches, and reported predictors, findings were synthesized narratively rather than quantitatively pooled. To improve interpretability, we grouped included studies according to study design, categorizing them as: (1) case-control studies (comparing individuals with suicidal ideation to those who had engaged in suicide attempts or other suicidal behaviors), (2) case-crossover studies, and (3) longitudinal studies (including prospective, retrospective, cohort, register-based, and EMA designs where SI was measured prior to subsequent suicidal behavior).

Within each design category, we further organized results by the type of risk factors examined for their association with transition from SI to SB. Risk factors were grouped based on conceptual similarity. When studies used different terminology to describe comparable constructs, risk factors were harmonized into shared categories to allow clearer comparison across studies. Findings were synthesized narratively, emphasizing patterns of evidence across studies and documenting differences in samples and design, with quantitative results reported when available and also described qualitatively.

### Risk of bias assessment

Risk of bias was assessed using the Newcastle-Ottawa Scale (NOS) for case-control and cohort studies [14]. Studies were rated (0–9) across standard domains of selection, comparability, and outcome/exposure assessment, with higher scores indicating lower risk of bias. One primary reviewer conducted the scoring, and a subset of studies was independently assessed by a second author, demonstrating high inter-rater reliability.

Across all included studies, NOS scores ranged from 2 to 8 out of a possible 9 points, indicating variable methodological quality (Table S3). The mean NOS score was 5.2, and the median score was 5, reflecting overall moderate study quality. Most studies scored relatively well in the comparability domain, with many receiving one or two stars for controlling key confounders. In contrast, variability was more apparent within the selection and outcome/exposure domains, where several studies did not meet all criteria. Overall, the distribution of scores suggests that most studies demonstrated moderate methodological rigor, with many meeting key quality criteria across domains. While some variability was observed in areas such as selection and measurement, the studies generally showed acceptable methodological standards. Taken together, the body of evidence can be interpreted with reasonable confidence, reflecting a solid overall level of study quality.

### Search results

**Fig. 1:**
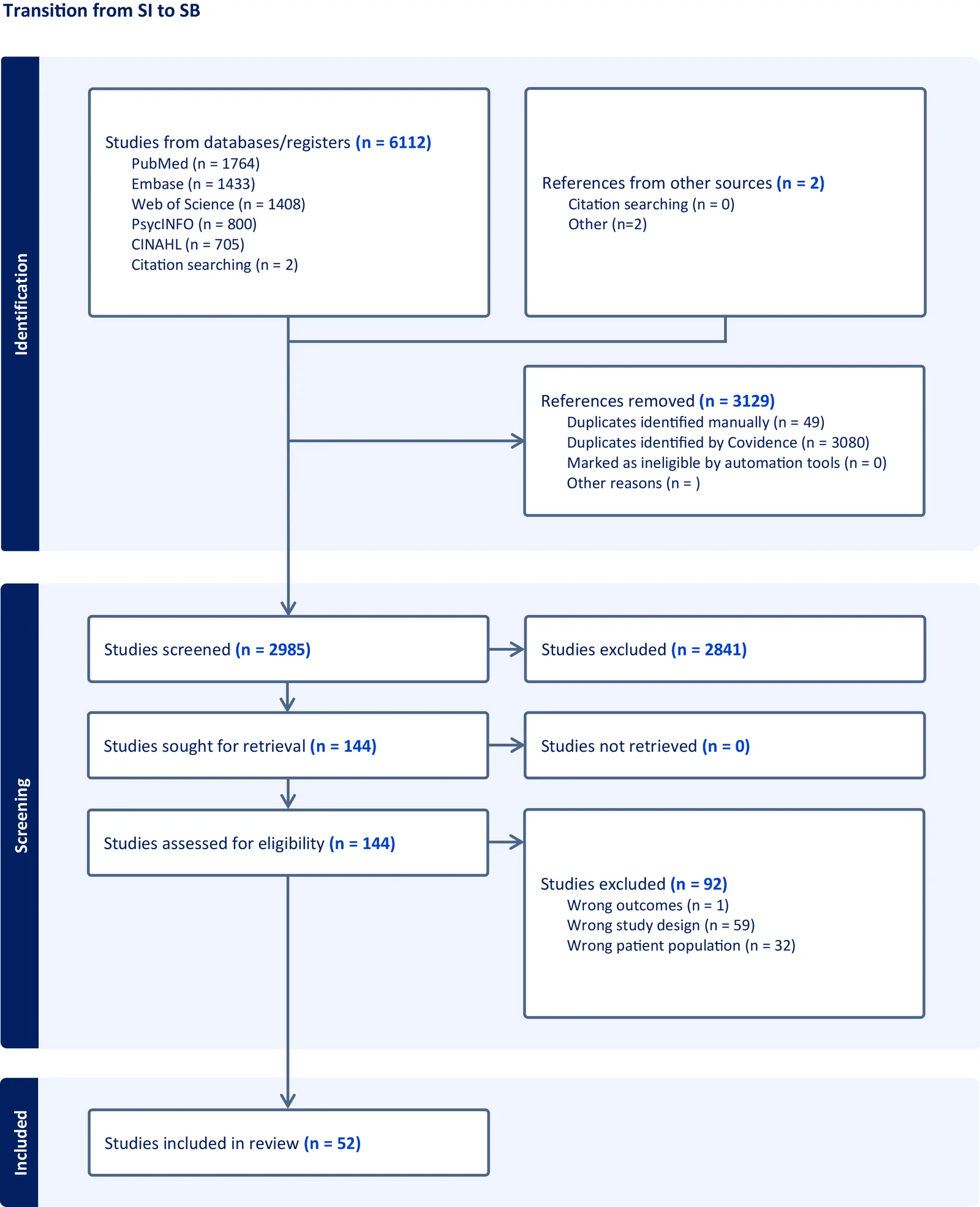
Study selection.

Across five databases, we identified 6112 records (PubMed *n* = 1764; Embase *n* = 1433; Web of Science *n* = 1408; PsycINFO *n* = 800; CINAHL *n* = 705) and 2 additional records through citation searching. After removing 3129 duplicates (49 manually; 3080 via Covidence), 2984 records were screened, and 2841 were excluded at the title/abstract stage. We sought full texts for 144 articles, all of which were retrieved and assessed for eligibility. Of these, 92 were excluded (wrong outcomes *n* = 1; wrong study design *n* = 59; wrong patient population *n* = 32), leaving 52 studies included in the review. (Fig. 1; Table 1).

**Table 1:** Characteristics of included studies.

Title	Authors	Study type	Year of publication	Country	Age range	Age mean	Sample size
Suicidal Thoughts and Behaviors Among Adolescent Psychiatric Inpatients	Alqueza KL; Pagliaccio D; Durham K; Srinivasan A; Stewart JG; Auerbach RP	Case-Control	2023	USA	12-19	15.58	970
Nightmares and Capability for Suicide: Implications in Adolescents at High-Risk for Suicide	Amadasu EE; Lange Valenga G; Dieste S; Gazor A; Wolfe K; Brown WD; Stewart SM	Case-Control	2025	USA	N/A	14.67	340
Measuring acquired capability for suicide within an ideation-to-action framework.	Burke TA; Ammerman BA; Knorr AC; Alloy LB; McCloskey MS	Case-Control	2018	USA	N/A	20.62	546
Risk Factors of Suicide Attempt among Adolescents with Suicide Ideation in Low- and Middle-Income Countries across the Globe	Ding L; Liu Y; Liu X	Case-Control	2023	39 LMICs	12-18	N/A	22,655
Externalizing behaviors, impulsivity, alexithymia, and emotional dysregulation in adolescents' suicidality	Gatta M; Raffagnato A; Angelico C; Benini E; Medda E; Fasolato R; Miscioscia M	Case-Control	2023	Italy	10-18	14.50	190
Rethinking Impulsivity in Suicide	E. David Klonsky; Alexis May	Case-Control	2010	USA	13–24	20.00	3706
Differentiating Adolescent Suicide Attempters and Ideators: A Classification Tree Analysis of Risk Behaviors	Alexis M. May; Ewa K. Czyz; Brady T. West	Case-Control	2020	USA	N/A	∼16	7493
Differentiating Adolescent Suicide Attempters from Ideators: Examining the Interaction between Depression Severity and Alcohol Use	Kimberly H. McManama O’Brien; Sara J. Becker; Anthony Spirito; Valerie Simon; Mitchell J. Prinstein	Case-Control	2014	USA	12-15	13.60	108
Blunted HPA axis activity prior to suicide attempt and increased inflammation in attempters	Nadine M. Melhem; Sara Munroe; Anna Marsland; Katarina Gray; David Brent; Giovanna Porta; Antoine Douaihy; Mark L. Laudenslager; Frank DePietro; Rasim Diler; Henry Driscoll; Priya Gopalan	Case-Control	2017	USA	15-30	23.00	115
Body Regard as a Volitional Factor for Suicide Attempts: Implications for Ideation to Action Frameworks	Kameron M. Mendes; Jennifer J. Muehlenkamp	Case-Control	2025	USA	N/A	19.52	2021
Differentiating Suicide Attempters from Suicide Ideators: The Role of Capability for Suicide	Yaxuan Ren; Jianing You; Xu Zhang; Jiyi Huang; Bradley T. Conner; Rui Sun; Sian Xu; Min-Pei Lin	Case-Control	2019	China	15–21	16.88	930
Violent victimization and perpetration as distinct risk factors for adolescent suicide attempts	Evan E. Rooney; Ryan M. Hill; Benjamin Oosterhoff; Julie B. Kaplow	Case-Control	2019	USA	13–21	16.20	821
Differentiating Suicide Ideators from Attempters: Violence-A Research Note	Steven Stack	Case-Control	2014	USA	N/A	16.10	2536
Risk and Protective Factors that Distinguish Adolescents Who Attempt Suicide from Those Who Only Consider Suicide in the Past Year	Lindsay A. Taliaferro; Jennifer J. Muehlenkamp	Case-Control	2014	USA	14–18	N/A	70,022
Hair cortisol concentrations as a putative biomarker for suicidal behavior	Lindsay Taraban; Emily Hone; Meilin Jia-Richards; Mary Ann Kelly; Jenna M. Lindsay; Sarah Riston; Zak Hutchinson; Thomas D. Walko III; Eli Goodfriend; Brian C. Thoma; Dara Sakolsky; Kehui Chen; Antoine Douaihy; David A. Brent; Anna L. Marsland; David A. Lewis; Nadine M. Melhem	Case-Control	2026	USA	18 = 30	24.66	238
Associations between impulsivity, aggression, and suicide in Chinese college students	Lin Wang; Chang Zhi He; Yun Miao Yu; Xiao Hui Qiu; Xiu Xian Yang; Zheng Xue Qiao; Hong Sui; Xiong Zhao Zhu; Yan Jie Yang	Case-Control	2014	China	16-43∗	21.30	479
Psychological Risk Factors in the Transition from Suicidal Ideation to Suicidal Behavior in Young Adults	Elif Yöyen; Merve Keleş	Case-Control	2024	Turkey	18–25	N/A	305
24-Hour warning signs for adolescent suicide attempts	King CA; Gipson Allen PY; Ahamed SI; Webb M; Casper TC; Brent D; Grupp-Phelan J; Rogers TA; Arango A; Al-Dajani N; McGuire TC; Bagge CL	Case-Crossover	2023	USA	13-18	15.0	1094
Exploratory analysis of mediators of the relationship between childhood maltreatment and suicidal behavior	Brooke A. Ammerman; Sarfaraz Serang; Ross Jacobucci; Taylor A. Burke; Lauren B. Alloy; Michael S. McCloskey	Prospective Cohort	2018	USA	11-21	15.15	4834
Using Smartphone GPS Data to Detect the Risk of Adolescent Suicidal Thoughts and Behaviors	Randy P. Auerbach; Paul A. Bloom; David Pagliaccio; Ranqing Lan; Hanga Galfalvy; Alma Bitran; Katherine Durham; Ryan Crowley; Karla Joyce; Ashley Blanchard; Lauren S. Chernick; Peter S. Dayan; Julia Greenblatt; Lauren E. Kahn; Giovanna Porta; Trinity C. Tse; Jeffrey F. Cohn; Louis-Philippe Morency; David A. Brent; Nicholas B. Allen	Prospective Case Series	2025	USA	13–18	16.4	186
Adolescent Predictors of Incidence and Persistence of Suicide-Related Outcomes in Young Adulthood: A Longitudinal Study of Mexican Youth	Corina Benjet; David Menendez; Yesica Albor; Guilherme Borges; Ricardo Orozco; Maria Elena Medina-Mora	Prospective Cohort	2018	Mexico	12-17, 19-26	N/A	1071
Predicting the Transition From Suicidal Ideation to Suicide Attempt Among Sexual and Gender Minority Youths	Johnny Berona, Ph.D., Sarah Whitton, Ph.D., Michael E. Newcomb, Ph.D., Brian Mustanski, Ph.D., Robert Gibbons, Ph.D.	Prospective Cohort	2021	USA	N/A	20.7	1006
Nicotine Dependence and Pre-Enlistment Suicidal Behavior Among U.S. Army Soldiers	Campbell-Sills L; Kessler RC; Ursano RJ; Sun X; Heeringa SG; Nock MK; Jain S; Stein MB	Retrospective Cohort	2019	USA	12-34	N/A	30,436
Childhood externalizing, internalizing and comorbid problems: distinguishing young adults who think about suicide from those who attempt suicide	Melissa Commisso; Caroline Temcheff; Massimiliano Orri; Martine Poirier; Marianne Lau; Sylvana Côté; Frank Vitaro; Gustavo Turecki; Richard Tremblay; Marie-Claude Geoffroy	Prospective Cohort	2021	Canada	6-22	N/A	2143
Predictive Validity of the Columbia-Suicide Severity Rating Scale for Short-Term Suicidal Behavior: A Danish Study of Adolescents at a High Risk of Suicide	Paul Maurice Conway; Annette Erlangsen; Thomas William Teasdale; Ida Skytte Jakobsen; Kim Juul Larsen	Prospective Cohort	2017	Denmark	<17	16.2	85
A Prospective Examination of the Interpersonal-Psychological Theory of Suicidal Behavior Among Psychiatric Adolescent Inpatients	Ewa K. Czyz; Johnny Berona; Cheryl A. King	Prospective Cohort	2015	USA	13–17	15.6	376
Longitudinal Trajectories of Suicidal Ideation and Subsequent Suicide Attempts Among Adolescent Inpatients	Ewa K. Czyz; Cheryl A. King	Prospective Cohort	2015	USA	13–17	15.6	376
HPA axis response and psychosocial stress as interactive predictors of suicidal ideation and behavior in adolescent females: a multilevel diathesis-stress framework	Tory A. Eisenlohr-Moul; Adam B. Miller; Matteo Giletta; Paul D. Hastings; Karen D. Rudolph; Matthew K. Nock; Mitchell J. Prinstein	Prospective Cohort	2018	USA	12-16	14.69	220
Fearlessness about death predicts adolescent suicide attempt: A preliminary analysis	Mikael S. Ferm; Laura A. Frazee; Betsy D. Kennard; Jessica D. King; Graham J. Emslie; Sunita M. Stewart	Prospective Cohort	2020	USA	12-18	14.86	122
Columbia-Suicide Severity Rating Scale Predictive Validity With Adolescent Psychiatric Emergency Patients	Polly Y. Gipson; Prachi Agarwala; Kiel J. Opperman; Adam Horwitz; Cheryl A. King	Prospective Cohort	2015	USA	13–17	15.25	178
Characteristics Differentiating Preadolescents With Suicidal Ideation From Those Who Display Suicidal Behaviors	Hennefield L; Donohue MR; Hoyniak CP; Whalen DJ; Thompson RJ; Tillman R; Luby JL; Barch DM	Prospective Cohort	2025	USA	8-12	10.12	195
Investigating the role of hallucinatory experiences in the transition from suicidal thoughts to attempts	Emily Hielscher; Jill DeVylder; Mark Connell; Penelope Hasking; Graham Martin; James G. Scott	Prospective Cohort	2020	Australia	12-17	13.9	216
Predicting Future Suicide Attempts Among Adolescent and Emerging Adult Psychiatric Emergency Patients	Adam G. Horwitz; Ewa K. Czyz; Cheryl A. King	Retrospective Cohort	2015	USA	15–24	19.38	473
Strengthening associations between psychotic like experiences and suicidal ideation and behavior across middle childhood and early adolescence	Nicole R. Karcher; Kirstie O’Hare; Samantha Y. Jay; Rebecca Grattan	Prospective Cohort	2023	USA	9-13	N/A	11,878
The associations between non-suicidal self-injury and first onset suicidal thoughts and behaviors	Kiekens, Hasking, Boyes, Claes, Mortier, Auerbach, Cuijpers, Demyttenaere, Green, Kessler, Myin-Germeys, Nock, Bruffaerts	Retrospective Cohort	2018	Belgium/Australia	N/A	18.9	6393
What distinguishes adolescents with suicidal thoughts from those who have attempted suicide? A population-based birth cohort study	Becky Mars; Jon Heron; E. David Klonsky; Paul Moran; Rory C. O’Connor; Kate Tilling; Paul Wilkinson; David Gunnell	Prospective Cohort	2019	UK	N/A	16.67	4772
Predictors of future suicide attempt among adolescents with suicidal thoughts or non-suicidal self-harm: a population-based birth cohort study	Becky Mars; Jon Heron; E David Klonsky; Paul Moran; Rory C O’Connor; Kate Tilling; Paul Wilkinson; David Gunnell	Prospective Cohort	2019	UK	16–21	N/A	456
Predicting suicidal events: A comparison of the Concise Health Risk Tracking Self-Report (CHRT-SR) and the Columbia Suicide Severity Rating Scale (C-SSRS)	Taryn L. Mayes; Thomas Carmody; A. John Rush; Karabi Nandy; Graham J. Emslie; Beth D. Kennard; Kathryn Forbes; Manish K. Jha; Jennifer L. Hughes; Jessica K. Heerschap; Madhukar H. Trivedi	Retrospective Cohort	2023	USA	12-18	14.90	539
Ethnic Variations of Trajectories in Suicide Ideation and Attempt: From Middle School to High School	Jahun Kim; Kenneth Pike; Elizabeth McCauley; Ann Vander Stoep	Prospective Cohort	2019	USA	12-18	12.02	463
Characteristics of suicidal ideation that predict the transition to future suicide attempts in adolescents	Regina Miranda; Ana Ortin; Michelle Scott; David Shaffer	Prospective Cohort	2014	USA	12-21	15.6	506
A longitudinal study of adolescent pathways differentiating suicide ideation and attempt in early adulthood	Geneviève Morneau-Vaillancourt; Massimiliano Orri; Isabelle Ouellet-Morin; Marie-Claude Geoffroy; Michel Boivin	Prospective Cohort	2025	Canada	12-23	N/A	1647
A Prospective Examination of the Predictive Validity of Three Transdiagnostic Assessments of Risk for Suicidal Behavior: Psychache, the Interpersonal Theory of Suicide, and Reasons for Living	Benjamin T. Paul, Catherine Greeno, Neal D. Ryan, Fuchiang (Rich) Tsui, Robert D. Gibbons, Giovanna Porta, Thomas Joiner, David Brent	Prospective Cohort	2025	USA	12-24	18.1	827
Prevalence, Correlates, and Treatment of Lifetime Suicidal Behavior Among Adolescents: Results From the National Comorbidity Survey Replication Adolescent Supplement	Matthew K. Nock; Jennifer Greif Green; Irving Hwang; Katie A. McLaughlin; Nancy A. Sampson; Alan M. Zaslavsky; Ronald C. Kessler	Retrospective Cohort	2013	USA	13-18	N/A	6483
Risk Factors for the Transition from Suicide Ideation to Suicide Attempt: Results from the Army Study to Assess Risk and Resilience in Servicemembers (Army STARRS)	Matthew K. Nock; Alexander J. Millner; Thomas E. Joiner; Peter M. Gutierrez; Georges Han; Irving Hwang; Andrew King; James A. Naifeh; Nancy A. Sampson; Alan M. Zaslavsky; Murray B. Stein; Robert J. Ursano; Ronald C. Kessler	Retrospective Cohort	2018	USA	N/A	17, 18 ∗	3916
Neurocognitive vulnerability to youth suicidal behavior	Donna Ruch; Arielle H. Sheftall; Kendra Heck; Sandra M. McBee-Strayer; Jaclyn Tissue; Brady Reynolds; John Ackerman; David A. Brent; John V. Campo; Jeffrey A. Bridge	Prospective Cohort	2020	USA	12-15	13.83	309
Adolescent Suicidal Ideation Subgroups and their Association with Suicidal Plans and Attempts in Young Adulthood	Martha A. Rueter; Kristen E. Holm; Christine R. McGeorge; Rand D. Conger	Prospective Cohort	2008	USA	14–27	N/A	552
Non-suicidal self-injury and suicidal ideation as predictors of suicide attempts in adolescent girls: A multi-wave prospective study	Lori N. Scott; Paul A. Pilkonis; Alison E. Hipwell; Kate Keenan; Stephanie D. Steppa	Prospective Cohort	2015	USA	10-21	N/A	1950
The relationship between parenting behavior, suicidal ideation, and suicide attempt across two population-based samples of adolescents	Mallory Stephenson; Jessica E. Salvatore; Séverine Lannoy; Alexis C. Edwards	Prospective Cohort	2025	USA	9-13, 12-21	9.47, 15.55	639, 978
Self-compassion and family cohesion moderate the association between suicide ideation and suicide attempts in Chinese adolescents	Ruohan Sun; Yaxuan Ren; Xiaoan Li; Yongqiang Jiang; Sihan Liu; Jianing You	Prospective Cohort	2020	China	N/A	12.96	520
The role of resilience in the transition of suicidal indicators among Chinese children and adolescents: a nested case-control study	Xin Tian; Linling Jiang; Shuqing Liu; Yandie He; Guiqing Zheng; Xinyi Liu; Xiang Wang; Yi Xiang; Jin Lu; Yuanyuan Xiao	Prospective Cohort	2025	China	10-17	13.56	5620
Do connectedness and self-esteem play a role in the transition to future suicide attempts among Latina and Latino youth with suicide ideation?	Ricardo J. Vélez-Grau; Michael A. Lindsey	Prospective Cohort	2022	USA	N/A	14.97	273
Suicide progression and influencing factors in Chinese adolescents: A latent transition analysis	Xuezhen Wang; Zhongjie Wang; Kaiyuan Lu; Juanjuan Zheng; Jingke He	Prospective Cohort	2024	China	13–15	13.61, 14.64	1407

Abbreviations: LMIC = Low and Middle Income Countries N/A = not applicable; UK = United Kingdom; USA = United States of America.

**Fig. 2:**
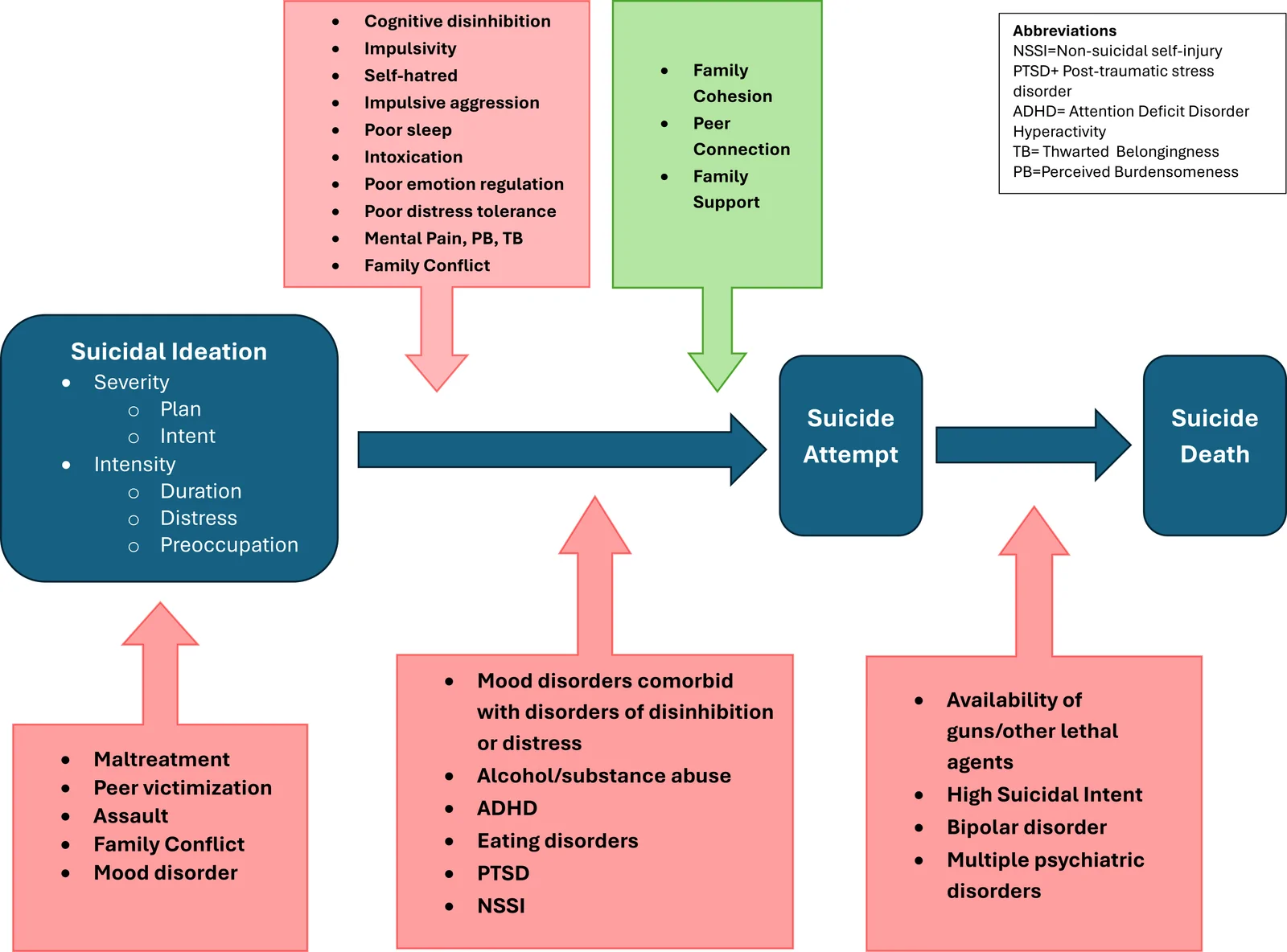
Risk and protective factors influencing the transition from suicidal ideation to suicidal attempt in youth.

## Results

In this section, we review case-control, retrospective, and prospective studies that describe the relationship between specific risk factors and the transition from suicidal ideation to suicide attempts and deaths. Table 2 summarizes the studies and findings for each category of risk or protective factor, with quantitative results when applicable. We then review studies that examined multiple risk factors simultaneously, to illustrate the relative contribution of different categories of risk factors. We conclude with a discussion of the strengths and limitations of the review and of the papers themselves, and the clinical implications, as well as areas for further research.

**Table 2:** Summary of identified risk factors and the supporting empirical evidence, including study type, sample characteristics, and key findings.

Abuse/Maltreatment exposure						
Study	Risk-factor-specific finding	Study type	Sample type	Country	Ages	Sample size
Ammerman (2018)	**Childhood maltreatment** predicted suicide attempts after SI among females (*b* = 0.19, 95% CI [0.04, 0.32])	Prospective Cohort	School-based sample	USA	11-21	4834
Wang (2024)	**Childhood maltreatment** predicted transition from SI to SA (OR = 2.615, 95% CI [1.146, 5.970]).	Prospective Cohort	School-based sample	China	13–15	1407
Alqueza (2021)	**Lifetime physical abuse** was associated with higher odds of SA (vs. SI-only), both unplanned (*aOR* = 2.67, 95% CI [1.31, 5.42]) and planned (*aOR* = 1.82, 95% CI [1.13, 2.95]) attempts.	Case-Control	Psychiatric inpatient sample	USA	12-19	970
Burke (2018)	SA (vs. SI-only) was associated with greater **childhood emotional abuse** (*d* = 1.19, *p* < 0.001), **childhood physical abuse** (*d* = 0.67, *p* < 0.001), and **physical neglect** (*d* = 0.53, *p* = .003).	Case-Control	College-based sample	USA	mean = 20.62	546
Taliaferro (2014)	Among females, **physical abuse** (OR = 1.66, 95% CI [1.28, 2.16]), **and sexual abuse** (OR = 1.43, 95% CI [1.12, 1.82]) were associated with higher odds of SA compared to SI-only.	Case-Control	Population-based sample	USA	14–18	70,022
**Stressful/Traumatic Life Events**						
Benjet (2018)	Greater **traumatic life events** predicted transition from SI to SA (*HR* = 1.48, 95% CI [1.09, 2.00]).	Prospective Cohort	Community-based sample	Mexico	12-17, 19-26	1071
Berona (2021)	**Victimization** predicted transition from SI to SA (*HR* = 2.48, 95% CI [1.10, 5.59]).	Prospective Cohort	Community-based sample	USA	mean = 20.7	1006
Stephenson (2025)	**Painful/provocative events** were associated with increased transition risk (*ORs* = 1.30–2.00).	Prospective Cohort	Population-based sample	USA	9-13, 12-21	639, 978
Hennefield (2025)	**Stressful events** (*OR* = 1.20) and **traumatic events** (*OR* = 1.82) distinguished SA from SI-only.	Prospective Cohort	Clinical preschool sample	USA	8-12	195
Morneau-Vaillancourt (2025)	Experiencing **peer victimization** (1%→2% probability increase) during adolescence differentiated ideators from attempters.	Prospective Cohort	Population-based sample	Canada	12-23	1647
May (2020)	**History of rape** was associated with a higher likelihood of SA compared to SI-only (58% vs. 29%).	Case-Control	School-based nonclinical sample	USA	mean = ∼16	7493
Rooney (2019)	Among adolescents with past-year SI, greater **violent victimization** was associated with more frequent SA (*β* = .13, *SE* = .06). The **victimization × perpetration** interaction was associated with more frequent SA (*β* = .11, SE = .03).	Case-Control	Population-based sample	USA	13–21	821
Taliaferro (2014)	**Dating violence victimization** was associated with higher odds of SA (vs. SI-only) among both males (OR = 1.51, 95% CI [1.07, 2.12]) and females (OR = 1.58, 95% CI [1.25, 1.99]).	Case-Control	Population-based sample	USA	14–18	70,022
**Exposure to Suicidal Behaviors**						
Mars (2019)a	**Exposure to self-harm in others** differentiated SA from SI-only, including family exposure (*OR* = 1.95), friend exposure (*OR* = 2.61), and combined family and friend exposure (*OR* = 5.26).	Prospective Cohort	Population-based sample	UK	mean = 16.67	4772
Mars (2019)b	**Exposure to self-harm** (family: OR = 2.03, 95% CI [0.93, 4.44]; friend: OR = 1.85, 95% CI [0.93, 3.69]) predicted subsequent SA among adolescents with SI.	Prospective Cohort	Population-based sample	UK	16–21	456
Alqueza (2021)	**Peer history of SA** was associated with higher odds of transition from SI to SA among ideators with a plan (*aOR* = 1.93, 95% CI [1.33, 2.80]).	Case-Control	Psychiatric inpatients	USA	12-19	970
Ding (2023)	**Close friends with SA history** was associated with higher odds of SA compared to SI-only among adolescents with SI (*OR* = 1.24, 95% CI [1.09, 1.42]).	Case-Control	School-based nonclinical sample	39 LMICs	12-18	22,655
**Family History of Mental Disorder**						
Benjet (2018)	Among baseline ideators, later SA was predicted by **parental mental illness** (*HR* = 7.68, 95% CI [1.56–37.80]).	Prospective Cohort	Community-based sample	Mexico	12-17, 19-26	1071
Yöyen (2024)	Being in the suicide attempt group (vs. ideation-only) was associated with **having a psychiatric diagnosis in the family** (*OR* = 1.901, 95% CI [1.122–3.220]).	Case-Control	Social media-based snowball sample	Turkey	18–25	305
Gatta (2023)	SA (vs. SI-only) was differentiated by **family history of psychiatric disorders**.	Case-Control	Psychiatric inpatients	Italy	10-18	190
**Non-Suicidal Self-Injury**						
Gipson (2015)	**Lifetime history of NSSI** predicted returning to psychiatric emergency services and SA at a return visit (return PE visit: *OR* = 2.19, 95% CI [1.09, 4.39]).	Prospective Cohort	Clinical sample	USA	13–17	178
Mars (2019)b	**Non-suicidal self-harm** predicted transition from SI to SA (*OR* = 2.78, 95% CI [1.35, 5.74]).	Prospective Cohort	Population-based	UK	16–21	456
Scott (2015)	For girls, **SI + NSSI status** was associated with higher odds of lifetime SA (*OR* = 12.63, 95% CI [5.91, 27.00]) and recent SA (*OR* = 4.43, 95% CI [1.74, 11.31]), whereas SI-only status was associated with more modest odds of lifetime SA (*OR* = 3.04, 95% CI [1.45, 6.38]).	Prospective Cohort	Low-icnome community-based sample	USA	10-21	1950
Stephenson (2025)	**NSSI** was associated with higher odds of SA among adolescents with SI (*OR* = 2.60, 95% CI [1.50, 4.60]).	Prospective Cohort	Population-based sample	USA	9-13, 12-21	639, 978
Kiekens (2018)	**NSSI** predicted transition from SI to SA (*OR* = 2.90, 95% CI [2.00, 4.30]).	Retrospective Cohort	College-based sample	Belgium/Australia	mean = 18.9	6393
Burke (2018)	Greater **frequency of NSSI** was reported among suicide attempters with intent to die compared to those without intent to die and those with a history of plans or ideation.	Case-Control	College-based sample	USA	mean = 20.62	546
Ren (2019)	**NSSI** differentiated adolescents who transitioned from SI to SA (*OR* = 6.26, 95% CI [1.02, 38.55]).	Case-Control	School-based sample	China	15–21	930
Taliaferro (2014)	For males, **self-injury** was associated with higher odds of SA compared to SI-only (*OR* = 5.17, 95% CI [3.93, 6.81]); for females, **self-injury** was also associated with higher odds of SA (*OR* = 3.90, 95% CI [3.05, 4.98]).	Case-Control	Population-based sample	USA	14–18	70,022
**Suicide Planning**						
Nock (2013)	**Having a suicide plan** was associated with higher odds of transition from SI to SA (*OR* = 5.30); 60.8% of ideators with a plan attempted suicide compared to 20.4% without a plan.	Retrospective Cohort	Nationally representative household and school sample	USA	13-18	6483
Stack (2014)	After adjustment for covariates, **having a suicide plan** differentiated SA from SI-only (*OR* = 2.69).	Case-Control	School-based nonclinical sample	USA	mean = 16.10	2536
**Baseline Suicidality/Ideation Measures**						
Conway (2017)	**Baseline suicide attempts** predicted short-term reattempts (*ORs* = 11.50–16.67), and **baseline suicidal behavior** predicted subsequent suicidal behavior of any type (*ORs* = 4.03–8.21). **Suicidal ideation severity** and **ideation with intent** predicted future suicidal behavior (*ORs* = 1.66–10.62), while **ideation intensity** incrementally predicted suicidal behavior (*ORs* = 1.27–1.30). The **ideation intensity components (frequency, duration, and deterrents)** predicted subsequent suicidal behavior and attempts (*ORs* = 1.92–3.12).	Prospective Cohort	Psychiatric outpatients	Denmark	<17	85
Czyz (2015)b	**Chronically elevated suicidal ideation trajectory** (predicted by higher baseline depressive symptoms, externalizing problems, and hopelessness) was associated with greater odds of subsequent SA (ORs = 2.29–4.15) and psychiatric rehospitalization (ORs = 3.23–11.20) compared to declining or subclinical ideation trajectories.	Prospective Cohort	Psychiatric inpatients	USA	13–17	376
Gipson (2015)	Greater **suicidal ideation intensity** at the index visit predicted subsequent SA at a return psychiatric emergency visit over 1-year follow-up (*OR* = 1.09, 95% CI [1.01–1.17]).	Prospective Cohort	Clinical sample	USA	13–17	178
Kim (2019)	**Suicidal ideation trajectory class** predicted later SA, with the “high-fluctuating ideators” class predicting SA among Asian American youth (*OR* = 10.0, 95% CI [1.28–78.12]); both “ideators” (*OR* = 11.56, 95% CI [1.84–72.73]) and “high-decreasing ideators” (*OR* = 25.69, 95% CI [5.88–112.34]) predicting SA among European American youth; and no significant prediction among African American youth.	Prospective Cohort	Community-based sample	USA	12-18	463
Miranda (2014)	**Frequent suicidal ideation** was associated with future SA (*OR* = 3.50, 95% CI [1.70–7.20]), **serious suicidal ideation** was associated with future SA (*OR* = 3.10, 95% CI [1.40–6.70]), and **long-duration suicidal ideation** was associated with future SA (*OR* = 2.30, 95% CI [1.10–5.20]). Among ideators, **ideation lasting ≥1 h (vs. <1 h)** was associated with future SA (*OR* = 3.60, 95% CI [1.00–12.70]) and earlier attempt onset, adjusting for covariates.	Prospective Cohort	Community-/school-based sample	USA	12-21	506
Paul (2025)	**Prior suicidal behavior** (*OR* = 5.43) predicted future SA.	Prospective Cohort	High risk clinical sample	USA	12-24	827
Rueter (2008)	**Increasing/worsening suicidal ideation trajectory** was associated with the highest later risk of suicide plans/attempts (plans: females r = .54, males r = .51; attempts: females r = .25), while male **decreasing ideation trajectory** was associated with the highest attempt probability among males (r = .36).	Prospective Cohort	Community-based sample	USA	14–27	552
Horwitz (2015)	**SI severity** (*OR* = 1.51, 95% CI [1.24–1.84]) and, among index-visit ideators, **SI intensity** (*OR* = 1.15, 95% CI [1.03–1.29]) were associated with transition from SI to SA.	Retrospective Cohort	Clinical sample	USA	15–24	473
Mayes (2023)	**CHRT-SR9 total score** (*OR* = 1.09, 95% CI [1.03–1.16]) and the **C-SSRS SI Intensity Composite** (*OR* = 1.16, 95% CI [1.02–1.31]), were associated with subsequent SA during treatment.	Retrospective Cohort	Psychiatric outpatients	USA	12-18	539
Nock (2018)	Among those with lifetime SI, **poor controllability of suicidal thoughts** was associated with higher odds of SA (*ORs* = 2.30–3.00).	Retrospective Cohort	Soldiers	USA	mean = 17	3916
**Suicide Screening Instruments**						
Berona (2021)	Among adolescents with SI, **baseline CAT-SS score** predicted subsequent SA (*HR* = 1.51, 95% CI [1.06, 2.15]).	Prospective Cohort	Community-based sample	USA	mean = 20.7	1006
Mayes (2023)	**CHRT-SR9 total score** (*OR* = 1.09, 95% CI [1.03–1.16]) and the **C-SSRS SI Intensity Composite** (*OR* = 1.16, 95% CI [1.02–1.31]), were associated with subsequent SA during treatment.	Retrospective Cohort	Psychiatric outpatients	USA	12-18	539
**Use of Drugs and Alcohol**						
Mars (2019)b	Among participants with SI, **cannabis use** (*OR* = 2.61, 95% CI [1.11–6.14]) and **other illicit drug use** (*OR* = 2.47, 95% CI [1.02–5.96]) predicted transition from SI to SA.	Prospective Cohort	Population-based	UK	16–21	456
Campbell-Sills (2019)	**Nicotine dependence** predicted transition from SI to unplanned SA (*aOR* = 2.03, 95% CI [1.36–3.03]).	Retrospective Cohort	Army soldiers	USA	12-34	30,436
Ding (2023)	Among adolescents with SI, **alcohol use** (*OR* = 1.70, 95% CI [1.56–1.86]) and **drug use** (*OR* = 1.80, 95% CI [1.56–2.08]) were associated with higher odds of SA compared to SI-only.	Case-Control	School-based nonclinical sample	39 LMICs	12-18	22,655
May (2020)	High transition risk among youth reporting **heroin use**.	Case-Control	School-based sample	USA	mean = ∼16	7493
McNamana O’Brien (2013)	**Higher alcohol use frequency** was associated with increased odds of recent SA (*OR* = 1.52, 95% CI [1.03–2.24]).	Case-Control	Psychiatric inpatients	USA	12-15	108
Stack (2014)	After adjustment for covariates, **cocaine use** differentiated SA from SI-only (*OR* = 2.34).	Case-Control	School-based nonclinical sample	USA	mean = 16.10	2536
Taliaferro (2014)	Among males, **cigarette smoking** was associated with higher odds of SA compared to SI-only (*OR* = 1.52, 95% CI [1.07–2.15]).	Case-Control	Population-based sample	USA	14–18	70,022
**Externalizing Behaviors**						
Benjet (2018)	Among baseline ideators, **behavioral disorders** predicted later SA (*HR* = 27.45, 95% CI [4.65–165.90]).	Prospective Cohort	Community-based sample	Mexico	12-17, 19-26	1071
Commisso (2023)	**Externalizing problems** (*OR* = 2.01, 95% CI [1.29–3.12]) and **comorbid internalizing and externalizing problems** (*OR* = 2.28, 95% CI [1.29–4.03]) differentiated adolescents who attempted suicide from those with SI-only.	Prospective Cohort	Population-based	Canada	6-22	2143
Mars (2019)a	SA (vs. SI-only) was differentiated by **behavioral disorder** (*OR* = 2.90).	Prospective Cohort	Population-based sample	UK	mean = 16.67	4772
Nock (2013)	**Intermittent explosive disorder** predicted planned attempts (*OR* = 4.20, 95% CI [1.70–10.00]); **conduct disorder** predicted attempts controlling for plan (*OR* = 3.20, 95% CI [1.60–6.30]) and unplanned attempts (*OR* = 8.00, 95% CI [3.50–18.30]); and **attention-deficit/hyperactivity disorder** predicted unplanned attempts (*OR* = 2.50, 95% CI [1.10–5.50]).	Retrospective Cohort	Nationally representative household and school sample	USA	13-18	6483
Nock (2018)	Among lifetime ideators, **intermittent explosive disorder** (*OR* = 1.40, 95% CI [1.10–1.80]) and **frequent extreme risk-taking** (*ORs* = 1.60–2.70) were associated with SA.	Retrospective Cohort	Soldiers	USA	mean = 17	3916
Gatta (2023)	Adolescents with SA (vs. SI-only) reported higher **externalizing problems**, specifically **rule-breaking behavior** and **conduct problems**.	Case-Control	Psychiatric inpatients	Italy	10-18	190
May (2020)	Youth reporting **past-year physical fights** had higher transition risk (44%) compared to 29% among those without these risk factors.	Case-Control	School-based nonclinical sample	USA	mean = ∼16	7493
Stack (2014)	After adjustment for covariates **fighting** (*OR* = 2.18) and **weapon carrying** (*OR* = 1.13), differentiated SA from SI-only.	Case-Control	School-based nonclinical sample	USA	mean = 16.10	2536
Yöyen (2024)	**Anger/impulsivity** was associated with higher odds of being in the SA group compared to SI-only (*OR* = 1.08, 95% CI [1.01–1.16]).	Case-Control	Social media-based snowball sample	Turkey	18–25	305
**Mood Disorder**						
Hennefield (2025)	Compared to SI-only, preadolescents with SA exhibited higher **depression** (*OR* = 1.52).	Prospective Cohort	Clinical preschool sample	USA	8-12	195
Mars (2019)a	SA (vs. SI-only) was differentiated by **depression** (*OR* = 3.63).	Prospective Cohort	Population-based sample	UK	mean = 16.67	4772
Kiekens (2018)	**Major depressive disorder (MDD)** (*OR* = 2.30, 95% CI [1.60–3.20]) and **mania** (*OR* = 1.70, 95% CI [1.10–2.60]) predicted transition from SI to SA.	Retrospective Cohort	College-based sample	Belgium/Australia	mean = 18.9	6393
Nock (2018)	Among lifetime ideators, **major depressive episode** was associated with higher odds of SA (*OR* = 1.40, 95% CI [1.10–1.80]).	Retrospective Cohort	Soldiers	USA	mean = 17	3916
Nock (2013)	**Major depressive disorder/dysthymia** predicted suicide planning (*OR* = 2.00, 95% CI [1.10–3.80]) and attempts controlling for plan (*OR* = 2.40, 95% CI [1.10–5.20]).	Retrospective Cohort	Nationally representative household and school sample	USA	13-18	6483
Gatta (2023)	Adolescents with SA (vs. SI-only) reported higher **total alexithymia**.	Case-Control	Psychiatric inpatients	Italy	10-18	190
Stack (2014)	After adjustment for covariates, **major depression** differentiated SA from SI-only (*OR* = 1.86).	Case-Control	School-based nonclinical sample	USA	mean = 16.10	2536
**Anxiety**						
Tian (2025)	Family support was protective against progression from SI to suicide planning among adolescents, minority youth, and those with baseline **anxiety**.	Prospective Cohort	School-based sample	China	10-17	5620
Hennefield (2025)	Compared to SI-only, preadolescents with SA exhibited higher **anxiety** (*OR* = 1.32).	Prospective Cohort	Clinical preschool sample	USA	8-12	195
Mars (2019)a	SA (vs. SI-only) was differentiated by **anxiety disorder** (*OR* = 2.20).	Prospective Cohort	Population-based sample	UK	mean = 16.67	4772
Ding (2023)	Among adolescents with SI, **anxiety** was associated with higher odds of SA compared to SI-only (*OR* = 1.54, 95% CI [1.40–1.70]).	Case-Control	School-based nonclinical sample	39 LMICs	12-18	22,655
**PTSD**						
Nock (2018)	Among lifetime ideators, **PTSD** was associated with higher odds of SA (*OR* = 1.30, 95% CI [1.00–1.70]).	Retrospective Cohort	Soldiers	USA	mean = 17	3916
**Eating Disorders**						
Nock (2013)	**Eating disorders** predicted attempts controlling for plan (*OR* = 4.50, 95% CI [1.80–11.20]) and planned attempts (*OR* = 5.30, 95% CI [2.00–14.00]).	Retrospective Cohort	Nationally representative household and school sample	USA	13-18	6483
**Psychotic symptoms**						
Hielscher (2020)	**Auditory hallucinatory experiences combined with psychological distress** predicted transition from SI to SA (*OR* = 9.58, 95% CI [1.61–56.98]); **psychological distress** alone was also associated with increased transition risk (*OR* = 4.88, 95% CI [0.98–24.46]).	Prospective Cohort	Community-/school-based sample	Australia	12-17	216
Karcher (2023)	**Psychotic-like experiences at baseline** predicted worsening SI and suicidal behavior over time, including transition from SI to suicidal behavior.	Prospective Cohort	Community-based sample	USA	9-13	11,878
**Sleep Disturbances**						
Amadasu (2025)	**Nightmares** were associated with transition to SA, including **nightmare frequency × SI** (*OR* = 1.21 [1.01–1.46]) and **nightmare severity × SI** (*OR* = 1.98 [1.00–3.92]).	Case-Control	Psychiatric outpatients	USA	mean = 14.67	340
**Impaired Cognitive Control**						
Tian (2025)	**Higher baseline resilience score** was associated with reduced odds of progression from SI to suicide planning (*OR* = 0.86, 95% CI [0.75–0.99]) and from SI to SA (*OR* = 0.82, 95% CI [0.69–0.96]).	Prospective Cohort	School-based sample	China	10-17	5620
Ruch (2020)	Among females, **Affective Go/No-Go commission errors (impulsivity)** were associated with future SA (*OR* = 1.03, 95% CI [1.00–1.06]), and among males, poorer **Spatial Working Memory strategy (executive functioning deficits)** was associated with future SA (*OR* = 1.28, 95% CI [1.04–1.58]).	Prospective Cohort	Clinical sample with MDD hx	USA	12-15	309
Klonsky (2010)	Compared to SI-only, SA was associated with **poor premeditation** (*p* < 0.03).	Case-Control	Soldiers, college students, H.S. students	USA	13–24	3706
Wang (2014)a	Compared to students with SI-only, those with a history of SA exhibited higher **cognitive impulsivity** (*p* < 0.01).	Case-Control	Nonclinical population-based university sample	China	16–43	479
**Maladaptive Self-Perception**						
Sun (2020)	**Higher positive self-compassion** weakened the association between transiton from SI to SA (*β* = −0.36), whereas **higher negative self-compassion** strengthened this association (*β* = 0.42).	Prospective Cohort	School-based sample	China	mean = 12.96	520
Hennefield (2025)	Compared to preadolescents with SI-only, those with SA exhibited higher **maladaptive guilt** (*OR* = 3.31) and **catastrophizing** (*OR* = 1.24).	Prospective Cohort	Clinical preschool sample	USA	8-12	195
King (2023)	In the 24 h prior to SA, **perceived burdensomeness** (*OR* = 1.97 [1.09–3.57]), **angry or hostile thoughts** (*OR* = 2.02 [1.09–3.75]), **self-hate** (*OR* = 5.16 [1.83–14.55]), **emotional pain** (*OR* = 2.48 [1.18–5.22]), and **surging or overwhelming emotions** (*OR* = 2.74 [1.28–5.86]) were associated with elevated risk.	Case-Crossover	Clinical sample	USA	13-18	1094
Mendes (2025)	Higher **body regard** was associated with lower odds of past-year SA (*OR* = 0.60, 95% CI [0.40–0.91]).	Case-Control	College-based nonclinical sample	USA	mean = 19.52	2021
Wang (2014)a	Compared to students with SI-only, those with a history of SA exhibited higher **self-oriented attack** (*p* < 0.001).	Case-Control	Nonclinical population-based university sample	China	16–43	479
Yöyen (2024)	Being in the suicide attempt group (vs. ideation-only) was associated with **perceived burden on others** (*OR* = 1.051, 95% CI [1.017–1.086]).	Case-Control	Social media-based snowball sample	Turkey	18–25	305
**Connectedness**						
Auerbach (2025)	Within-person increases in **weekly homestay** predicted next-week suicidal events such that a 1-SD increase above one’s mean was associated with nearly double the odds of a suicidal event (*aOR* = 1.99, 95% CI [1.15–3.45], *p* = .01); **weekly homestay** was also associated with same-week suicidal events (*aOR* = 1.80, 95% CI [1.05–3.19]) and same-week clinically meaningful ideation (*aOR* = 1.47, 95% CI [1.04–2.08]).	Prospective Case Series	High risk convenience sample	USA	13–18	186
Sun (2020)	Higher **family cohesion** weakened the association between transition from SI to SA (*β* = −0.20).	Prospective Cohort	School-based sample	China	mean = 12.96	520
Tian (2025)	**Family support** was protective against progression from SI to suicide planning among adolescents, minority youth, and those with baseline anxiety.	Prospective Cohort	School-based sample	China	10-17	5620
Vélez-Grau (2022)	Higher **family connectedness** was associated with reduced odds of transitioning to SA relative to no ideation (*OR* = 0.14).	Prospective Cohort	School-based sample	USA	mean = 14.97	273
King (2023)	In the 24 h prior to SA, **withdrawal from people or activities** was associated with markedly increased odds of attempt in case-crossover analyses (*OR* = 15.44, 95% CI [2.68–89.04]). In the 24 h preceding SA, **serious parent–child conflict** also predicted attempts (OR = 10.97 [2.02–59.65]).	Case-Crossover	Clinical sample	USA	13-18	1094
Ding (2023)	Among adolescents with SI, **loneliness** was associated with higher odds of SA compared to SI-only (*OR* = 1.46, 95% CI [1.34–1.60]).	Case-Control	School-based nonclinical sample	39 LMICs	12-18	22,655
Taliaferro (2014)	For males, SA (vs. SI-only) was differentiated by higher **parent connectedness** (*OR* = 0.41 [0.24–0.72]); for females, higher **parent connectedness** was also associated with SA (*OR* = 0.28 [0.18–0.45]).	Case-Control	Population-based sample	USA	14–18	70,022
**Altered Stress Response**						
Eisenlohr-Moul (2018)	Higher-than-usual **peer stress** predicted suicidal ideation, and the **peer stress × low cortisol interaction** predicted suicidal behavior (attempt/aborted/interrupted) among girls with blunted cortisol reactivity.	Prospective Cohort	Clinical sample	USA	12-16	220
Melhem (2017)	Suicide attempters exhibited **lower pre-attempt HPA-axis activity**, **higher inflammation**, and **lower hair cortisol concentrations (HCC)** compared to both suicidal ideators and healthy controls.	Case-Control	Psychiatric inpatients	USA	15-30	115
Taraban (2026)	**Hair cortisol concentration** was significantly lower in individuals who died by suicide compared to those with a suicide attempt [Diff (SE) = −0.64 (0.20), *p* = 0.010, *d* = 0.73], suicidal ideation [Diff (SE) = −0.89 (0.20), *p* < 0.001, *d* = 1.02], and psychiatric comparison individuals [Diff (SE) = −0.74 (0.26), *p* = 0.022, *d* = 0.86], and each unit decrease in HCC was associated with increased odds of greater suicidal severity [OR = 0.66, 95% CI (0.52, 0.84), *p* = 0.001].	Case-Control	Psychiatric inpatients	USA	18–30	238
**Suicide Model Constructs**						
Czyz (2015)a	At 3 months, SA was predicted by low **AC** and higher **TB** (*OR* = 2.18, 95% CI [1.10–4.33]); at 12 months, high **AC** independently predicted SA (*OR* = 1.38, 95% CI [1.01–1.88]).	Prospective Cohort	Psychiatric inpatients	USA	13–17	376
Ferm (2020)	Higher **fearlessness about death (acquired capability)** at baseline predicted increased risk of suicide attempt during treatment or within 6 months post-discharge.	Prospective Cohort	Psychiatric outpatients	USA	12-18	122
Paul (2025)	Future SA was predicted by **psychache** (*ORs* = 1.06–1.07), **perceived burdensomeness** (*OR* = 1.07–1.08), **thwarted belongingness** (*OR* = 1.05), **acquired capability** (*ORs* = 1.07–1.10), and fewer **reasons for living** (*OR* = 0.98); **acquired capability** remained the only theory-based predictor independently associated with attempts after adjustment (AUROCs ≈ 0.81–0.84).	Prospective Cohort	High risk clinical sample	USA	12-24	827
Wang (2024)	**Psychache** predicted progression from SI to SA (*OR* = 3.052, 95% CI [1.268–7.343]).	Prospective Cohort	School-based sample	China	13–15	1407
Yöyen (2024)	SA (vs. SI-only) was predicted by **perceived burdensomeness** (*OR* = 1.051, 95% CI [1.017–1.086]) and **acquired suicide efficacy/death fearlessness** (*OR* = 1.050, 95% CI [1.023–1.078]).	Case-Control	Social media-based snowball sample	Turkey	18–25	305
**Received Mental Health Support**						
Yöyen (2024)	Being in the suicide attempt group (vs. ideation-only) was associated with **receiving psychological support** (*OR* = 2.090, 95% CI [1.048–4.168]).	Case-Control	Social media-based snowball sample	Turkey	18–25	305
**School Stressors**						
Morneau-Vaillancourt (2025)	SA (vs. SI-only) was differentiated by **unusual rises in school difficulties** during adolescence (probability increasing from 1% to 5%).	Case-Control	Population-based sample	Canada	12-23	1647
Taliaferro (2014)	For males, **higher GPA** was associated with lower odds of SA (vs. SI-only) (*OR* = 0.49 [0.31–0.78]); for females, **higher GPA** was also associated with lower odds of SA (*OR* = 0.30 [0.20–0.44]).	Case-Control	Population-based sample	USA	14–18	70,022
**Female Gender**						
Benjet (2018)	Among baseline ideators, later SA was predicted by **female sex** (*HR* = 13.20, 95% CI [2.17–80.16]).	Prospective Cohort	Community-based sample	Mexico	12-17, 19-26	1071
Yöyen (2024)	SA (vs. SI-only) was associated with **being female** (*OR* = 1.697, 95% CI [1.006–2.862]).	Case-Control	Social media-based snowball sample	Turkey	18–25	305

Abbreviations: AC=Acquired Capability; aOR=Adjusted Odds Ratio;CATSS= Computerized Adaptive Test-Suicide Screen; CHRT-SR-9=Concise Health Risk Tracking, Self-report, 9-item; CI=95% Confidence Interval; C-SSRS=Columbia Suicide Severity Rating Scale; GPA=Grade Point Average; HR=Hazard Ratio; NASI=Non-suicidal self-injury; OR=Odds Ratio; PE=Psychiatric Emergency Room; SA=Suicide Attempt; SI=Suicidal Ideation; TB=Thwarted Belonging.

### Distal and proximal stress and protective factors

Abuse/maltreatment. Case-control studies reported higher rates of physical abuse, neglect, sexual abuse, and broader category of child maltreatment in suicide attempts (SA) compared to suicidal ideators [[15], [16], [17]]. Prospective studies were generally convergent, showing that child maltreatment predicted a transition from SI to SA [18,19].

Stressors/assault/victimization. Case-control studies found that a history of rape and violent victimization, particularly dating violence differentiated SA from SI [17,20,21]. Those who were both peer victims and victimizers were also more commonly found among SA than SI [21]. Higher levels of school stressors and peer victimization also differentiated adolescent SA from SI [22]. Prospective studies show higher rates of peer victimization and traumatic events in those who transition from SI to SA [[23], [24], [25], [26]].

Family and peer connection and conflict. Case-control studies found that youth with SA had lower connectedness to parents than youth with SI [17]. Prospective studies showed that family connection, perceived family support, and family cohesion all protected against SI transitioning to SA [[27], [28], [29]]. In a case-control follow-back study, parent-child conflict was more common in the 24 h prior to the SA than in the preceding 24 h [30]. In this same study, King et al. also found that social withdrawal was a common imminent precursor of an SA. Additionally, another study found that loneliness also was more prominent in SA than in SI [31]. Convergent with King et al.’s finding about social withdrawal being an imminent indicator of suicidal risk were the findings of a longitudinal study of adolescents enriched for psychopathology and SI. These youth were followed for 6 months with via mobile sensing and found that increasing homestay was an imminent predictor of a suicide event (suicide attempt, visit to emergency department or hospital for suicidality) within a week of the increase in homestay [32]. Another study found that peer-victimization was also a risk factor for a suicide attempt, even adjusting for baseline severity of SI [33].

Exposure to suicidal behavior. Those who engaged in SA were much more likely than those with SI-only to have been exposed to a friend or family member’s SA. Having a peer or close friend who had made an SA differentiated SA from SI [31,34]. Prospective studies showed that exposure to both peer and familial suicidal behavior predicted a transition from SI to SA [35,36].

### Past and current suicidal ideation, suicidal behavior and non-suicidal self-injury

Characteristics of suicidal ideation. In a large epidemiological study, retrospective reconstruction of the course of SI and SA found that SI accompanied by a plan made a transition to an SA much more likely than did SI without a plan [6]; a history of ideation with a plan vs without a plan also differentiated SI from SA [37]. One prospective study [38] and one retrospective study [39] are consistent with these results, finding that suicide severity (higher severity associated with SI with intent or plan) increased the likelihood of a transition from SI to SA. Several longitudinal studies found that the intensity of SI (e.g., duration, chronicity, intrusiveness, lack of deterrents) was associated with the transition from SI to SA [[38], [39], [40], [41], [42], [43], [44]].

Past suicidal behavior. A prospective study of youth presenting to emergency departments found that controlling for recent suicidal ideation, a history of a suicide attempt was still a risk factor for a subsequent attempt [45]. A retrospective cohort study that examined PHQ-9M predictors of subsequent suicide attempt as represented in medical records found that past suicide attempt was a much stronger predictor of a subsequent suicide attempt than the presence of SI, even after controlling for the severity of suicidal ideation: Adjusted Hazard Ratio (AHR) = 3.08 (2.47–3.80) vs. 1.27 (0.96–2.45) [46].

Non-suicidal self-injury (NSSI). In three case-control studies, NSSI differentiated those who had engaged in SA vs. SI [16,17,47]. The frequency of NSSI also differentiated those who made an SA with vs. without intent, and those who had engaged in suicidal planning vs. those who had not. Several prospective and retrospective studies have shown that NSSI is a consistent predictor of the transition from SI to SA [26,36,41,48,49].

### Family and personal history of psychopathology

Family history of psychiatric disorder and suicidal behavior. In both case-control [50,51] and prospective studies, parental psychiatric disorder either differentiated SI from SA, or predicted this transition [23]. Studies that focused more exclusively on the role of family history of suicidal behavior found that suicidal behavior was more common in the parents of adolescent suicide decedents and adolescent SA than in community and psychiatric controls, respectively [52,53]. Parental history of an SA also predicted future suicide attempts in adolescent offspring, mediated by the transmission of impulsive aggression and depression [54]. A meta-analysis showed an association of parental suicide and suicide attempts with similar outcomes in offspring [55].

Alcohol and Substance abuse. In case-control studies, tobacco, cocaine, heroin, cannabis, other drug use, and alcohol use were all more common in SA vs. SI [17,20,31,37,[56], [57], [58]]. A large retrospective cohort study found that both tobacco and alcohol use were associated with the transition from SI to SA [6]. A large community longitudinal study found that cannabis and illicit drug use were both predictors of the transition from SI to SA [36].

Externalizing disorders. In a a retrospective cohort study, the presence of intermittent explosive disorder and conduct disorder predicted transition from SI to SA, and the presence of attention deficit hyperactivity disorder (ADHD) predicted a transition from SI to an unplanned SA [6]. Case-control studies are consistent with this finding: higher rates of externalizing disorder, physical fighting, violent behavior, anger and impulsivity, externalizing disorder alone, or externalizing disorder comorbid with internalizing disorder all differentiate SA from SI [20,37,50,51,59]. One prospective study found that externalizing disorders were strongly predictive of the transition from SI to SA [23]. Impulsive aggression makes a stronger contribution to suicide in younger persons than in older people [60]. A retrospective study of soldiers [44] found that intermittent explosive disorder, and tendency to risk-taking were predictors of the transition from SI to SA.

Depression. Studies show higher rates of depression in pre-adolescent [25] and adolescent SA vs. SI [35,37]. In a retrospective cohort study, Nock et al. found that depression predicted the transition from SI without a plan to SI with a plan, and to SA [6]. Kiekens found in a retrospective study that depression and mood symptoms were significant predictors of the transition from SI to SA [49].

Anxiety and Post-traumatic stress disorder (PTSD). Prospective [25,35] and case-control studies [31] in pre-adolescent and adolescent youth find that anxiety is associated with the transition from SI to SA. Nock et al. 2018 in a prospective study of soldiers found that PTSD predicted a transition from SI to SA [44].

Eating disorders. In a retrospective cohort study, Nock et al. (2013) found that eating disorders predicted the transition from ideation with planning to SA, and from ideation to a planned SA in adolescents [6].

Psychotic symptoms. Auditory hallucinations, especially when accompanied by psychological distress, predicted a transition from SI to SA, as did the broader category of psychotic symptoms [61,62].

Sleep. One case-control study found that nightmare frequency and severity predicted a transition from SI to SA [63]. A prospective study of soldiers with SI using administrative data found that being diagnosed with a sleep disorder at the time of presentation for SI predicted a transition to an SA [64].

Cognitive control. Case-control and prospective studies find that SA, compared to SI, showed an impaired ability to consider future consequences [65], disrupted cognitive inhibition, impaired working memory [66], and cognitive impulsivity [67].

Emotion regulation and distress intolerance. In a case-control study of soldiers, less adaptive emotion regulation and distress tolerance differentiated SA from those who had experienced SI in the past 12 months [68].

Views of self. Case-control studies show that SA, compared to SI have a more negative body image [69], self-hate [67], and view of self as being burdensome to other [50]. A prospective study found that maladaptive guilt significantly predicted transition from SI to SA [25]. King et al. (2023) conducted a case-control follow-back study, in which the rates of occurrence of risk factors in the 24 h prior to the suicide attempt were compared to those in the 48 h before. Feelings of burdensomeness and self-hate were more common in the 24 h leading up to the attempt [30]. Conversely, self-compassion was protective against SI transitioning to an SA [27].

Altered stress response. Einsenlohr-Moule and colleagues conducted a longitudinal study of adolescent girls and found that high levels of social stress resulted in an increase in SI, however, high social stress only resulted in SA among girls who previously exhibited a blunted cortisol response to social stress on the Trier Social Stress [70]. Consistent with these findings, Melhem et al. compared cortisol responses to the Trier Social Stress Test in offspring of parents with mood disorders and found that blunted cortisol reactivity was associated with a history of a suicide attempt, particularly in those with a family history of a suicide attempt [71]. O’Connor and colleagues, using a similar cortisol reactivity paradigm, the Maastrecht Acute Stress Test, also found blunted cortisol reactivity associated with a history of a suicide attempt [72].Similar to the findings of Melhem et al., blunted cortisol reactivity was most prominent in those with a history of a suicide attempt who also had a family history of suicide behavior.

The papers of O’Connor and of Melhem correlate blunted cortisol response with a history of a suicide attempt, so that it is not possible to determine if the blunted cortisol reactivity preceded the suicide attempt, or was a consequence of it. The study of Eisenlohr-Moule et al. does suggest that a blunted cortisol response to stress could be predictive of a future suicide attempt, although the prediction was in a sample of adolescent females and was also conditional on the experience of social stress.

One approach to determine if altered cortisol dynamics precede a suicide attempt is through the use of hair cortisol concentration (HCC). If the hair is sampled within an inch of the scalp, the cortisol concentration in the hair gives an average estimate of the levels of cortsol in the previous month,. Blunted hair cortisol associated with a recent suicide attempt would support the view that blunted cortisol response antedates, and may predispose to future suicidal behavior.

In a sample of adolescent and young adult patients with SI, SA, or who were non-suicidal psychiatric controls (PC), hair cortisol (HCC) was lower in SA than in SI or PC [73]. In a more recent study, Taraban and colleagues showed that young adult suicide decedents had a lower HCC than young adults with SI and PC, but not SA [74]. Therefore, these findings support the view that blunted cortisol response to stress and secretion are markers that predict an increased likelihood of a suicide attempt.

Measures of suicidal risk. The K-CAT-SS is a computerized adaptive test designed to predict future SI and SA. In a longitudinal study of nearly 700 high-risk adolescents and young adults (78% of whom had had recent SI), the K-CAT-SS predicted an SA with high accuracy (Area Under the Response Operator Curve [AUROC] = 0.83). The K-CAT-SS continued to be a strong predictor even after adjusting for age, race, ethnicity, sex, sexual orientation, gender identity, and history of past SA [75]. The Computerized Adaptive Screen for Suicidality in Youth (CASSY) was developed in the ED-STARS study, a multi-site study in pediatric EDs that examined the risk and course of SI and SA [76]. The predictive validity of the CASSY was compared to the performance of the ASQ, a widely utilized 4-item screen for suicidal risk. The sample, drawn from patients presented to pediatric EDs, was enriched for patients who made behavioral health visits, and around 2/3 of the sample had experienced recent SI. For youth presenting with medical complaints, the AUROC for the ASQ (0.88) and of the CASSY (0.94) for the prediction of SA within 3 months were similarly high. The CASSY was more accurate than the ASQ for those presenting with behavioral health complaints (AUROC’s 0.72 vs. 0.57) [77].

The Columbia Suicide Severity Rating Scale. The Columbia Suicide Severity Rating Scale, originally designed as an interview, can also be used as a self-report measure. The is a 6-item screening, and a more extensive interview that covers severity of suicidal ideation (on a spectrum from no ideation to ideation with plan and intent), suicidal intensity (pre-occupation, ability to distract oneself from thoughts of suicide), non-suicidal self-injury (thoughts, lethality, frequency, motivation), and past suicidal behavior, which includes behavior preparatory to suicidal behavior, an aborted, interrupted, or actual suicide attempt, and the degree of planning, intent, and lethality associated with the behavior. A meta-analysis that covers the entire age spectrum but includes adolescents and young adults found that suicidal ideation severity, and to a lesser extent intensity predicted future suicide attempts, as did past suicidal behavior [78]. Conway (2016) in a prospective study of 85 adolescents, found that past attempts and ideation intensity predicted future suicide attempts, and that ideation severity predicted future attempts even after controlling past attempts [38]. Gipson, in a follow-up of 178 adolescents, found that suicide intensity, particularly duration, predicted a future attempt, even in those with current ideation [41]. A longitudinal study conducted in the Democratic Republic of Korea, 23.1% of which were adolescents, found that the severity of suicidal ideation and intensity of suicidal ideation was related to future suicidal behavior with AUROCs of 0.712 and 0.688, respectively [79].

The Concise Health Risk Tracking Form-Self Report (CHRT-SP) is a 14-item measure that assesses suicidal ideation, despair, helplessness, social support, and impulsivity. In a follow-up study of adolescent patients treated in an intensive outpatient program, this measure showed high sensitivity and moderate sensitivity for the prediction of suicide attempts and suicidal events (suicide attempts or visits to the ED for suicidality) [80].

The PHQ-9 and the PHQ-9M are two screening measures for depression that have become widely used in pediatric primary care. The PHQ-9 has one suicide-related item that asks about the frequency of thoughts of suicide and self-harm. The PHQ-9M has four additional items, namely, recent suicidal ideation, past suicide attempts, current impairment, and chronicity and pervasiveness of depression. The PHQ-9 item 9 has been shown to predict suicide attempts, with the odds ranging from 2.8 for having ideation a few tunes to 6.2 for those having ideation nearly every day, although 32% of those who make a suicide attempt within 30 days of screening deny suicidal ideation at screening. In a comparison of the PHQ-9 and the PHQ-9M, the latter was more accurate in predicting suicide attempts over the course of 365 days AUROC = 0.80 vs. 0.77). The adjusted odds for the suicide attempt item (only found in the PHQ-9M, 3.06)). was much higher than the suicide ideation item on the PHQ-9 (and 9 M) of 1.27 [81,82].

Theories of suicidal behavior. The Interpersonal Theory of Suicide (IPTS) is the most widely studied suicide-related theory [83]. It posits that perceived burdensomeness (PB) and thwarted belongingness (TB) were related to SI, which then, in the presence of acquired capability (AC), leads to SA. In several studies, proxies for AC were used, such as previous painful experiences, previous attempts, and NSSI [84]. In prospective and case-control studies, AC differentiated SA from SI [50,85,86]. One longitudinal study of youth at high risk for suicidal behavior (78% had recent history of SI) found that prospectively, TB, PB, and AC were all related prospectively to SA [87].

Another theory of suicidal behavior was advanced by Edwin Shneidman, termed “Psychache,” based on the view that suicidal behavior ensures when the suicidal person is faced with unbearable psychological pain [88]. In the above-noted longitudinal study, Psychache was a predictor of subsequent SA [87].

Transition for suicidal ideation to suicide death. A case-control study conducted in active-duty soldiers compared suicide decedents to propensity-matched soldiers with a history of SI within the past 12 months. The two groups were quite similar except that suicide decedents were much more likely to meet criteria for 3 or more psychiatric diagnoses within 30 days of their death [57]. A case-control study among Dutch adolescents matched suicide decedents to psychiatric controls with either suicidal ideation or suicidal behavior. The suicide decedents were less likely to have communicated their suicidal plans, had more exposure to suicidal behavior from peers, had more relationship problems, and were less likely to have received mental health treatment [89]. Another case-control study compared adolescent suicide decedents with patients hospitalized for suicidal ideation or an attempt. Suicide decedents were more likely than suicidal adolescents to have a diagnosis of bipolar disorder, a mood disorder with non-mood comorbidity, and lack of prior mental health treatment. Furthermore, suicide decedents, when compared to living attempters, showed higher suicidal intent and were more likely to have access to a gun [90].

One longitudinal study of suicide deaths in the Army STARRS study found that, in addition to a previous attempt while in the service, any suicidality, history of psychiatric hospitalization, outpatient specialty mental health care, non-affective psychosis, and bipolar disorder were predictors of suicide death [91]. Although not all of the participants in the study were suicidal, the study did control for current and past suicidality, thereby identifying those variables above and beyond SI that contributed to suicidal risk (i.e., non-affective psychosis, bipolar disorder, history of psychiatric hospitalization and/or outpatient mental health specialty care). A meta-analytic synthesis of case-control and longitudinal studies found that suicidal ideation and/or behavior conveyed a high risk for suicide of OR = 22.53 [92].

Multivariate prediction of SA. Although it is useful to see studies’ documentation of individual risk factors for SA, these studies, as presented, do not shed light on what are the most *salient* predictors of SA among suicidal patients. King et al., in the ED-STARS study of suicidal behavior in adolescents who present in pediatric EDs, identified predictors of an SA in patients seen in EDs, 66.3% of whom had had SI within the previous week [44,45]. Controlling for recent SI, additional predictors of an SA within the next 3 months were: lifetime severity of SI (e.g., ideation with intent and plan), lifetime history of suicidal behavior, and low school connection. The logistic regression with these variables included predicted future SA accurately (AUROC = 0.86).

Nock et al. (2018) reported on a longitudinal study of soldiers with SI, identifying the most parsimonious set of variables that predicted an eventual SA. Six variables were identified that together predicted a future SA with a very high AUROC = 0.93. The salient variables, and the population attributable risk proportion (PARP) were: SI with a plan (47.2%), mental disorder (e.g., depression, etc.; 27.5%); duration of SI (8,8%), controllability of SI (38.6%), risk-taking referred to as “tempting fate,” (37.8%), and NSSI (1%) [44].

## Discussion

In this systematic review of the factors that lead to the transition from suicidal ideation to suicidal behavior in youth, we found that maltreatment, exposure to traumatic events, severity and intensity of suicidal ideation, non-suicidal self-injury, most psychiatric conditions, family conflict and lack of parent-child connection, and negative views of self were consistent contributors to this transition (see Fig. 2). In the few studies that looked at these factors simultaneously, the most significant contributors were characteristics of suicidal ideation, namely the extent to which ideation was accompanied by a plan and/or suicidal intent, lack of controllability, and high duration and intensity. These aspects of suicidal ideation were the strongest predictors of a transition from ideation to suicidal behavior, even more so that psychopathology. There was also support for alterations in the hypothalamic-pituitary adrenal axis (HPA) that antedated and predisposed to suicidal behavior, especially in the face of social stress. Conversely, perceived connection and support were protective against such a transition. There was also support for theories of suicide that posited that mental pain, thwarted belongingness, perceived burdensomeness, and acquired capability for engaging in suicidal behavior were3 contributors to the transition from SI to SA.

The primacy of the severity and intensity of SI as predictors of future SA underscores the importance of conducting a careful assessment of relevant dimensions of SI in the suicidal patient. Specifically, the presence of a plan and/or intent, the intrusiveness, lack of controllability, and duration of ideation all contribute to an increased likelihood of a transition to suicidal behavior. Effective short-term interventions, such as safety planning depend on the ability of the patient to learn how to tolerate and moderate distress, engage in distress, and regulate response to mental pain through distraction or other types of adaptive coping, including connecting with relevant supportive individuals. In addition, NSSI, particularly of high frequency, has been shown to be a strong predictor of future suicidal behavior. Whether NSSI is simply a marker of poor emotion regulation, or whether engagement in self-destructive behavior makes the individual more likely to be willing to self-harm with suicidal intent is unclear, although the latter formulation has been posited by proponents of the IPTS. However, in either case, NSSI should be a treatment target, since the same set of skills and processes that enable a person to deter engaging in NSSI also will be protective against SA, as has been demonstrated in clinical trials of dialectic behavior therapy [93,94].

Sleep emerges as a very salient target to prevent suicidal behavior. Sleep-deprived youth are more impulsive, more reactive to negative social stimuli, and less likely to response to psychotherapy or antidepressant treatment of depression [95,96]. Conversely, the treatment of sleep difficulties can be protective against suicidal behavior. This is an important area of inquiry as sleep problems are ubiquitous among adolescents, a strong driver of suicidal ideation and behavior, and treatable [96].

The constellation of social stressors that contribute to suicidal risk range from peer victimization and family conflict to exposure to dating violence, rape, and maltreatment. Assessment of trauma should be a critical part of the ascertainment for suicidal risk, because the treatment of patients with post-traumatic stress disorder can result in significant reductions in suicidal risk [97]. In another study of safety planning, the presence of bipolar disorder was a negative moderator of outcome [98]. Therefore, the assessment of psychiatric conditions is essential because their remediation could be protective against suicidal behavior. In fact, eating disorders, depression, substance abuse, PTSD, and ADHD have all been reported to be “accelerators” of ideation to suicidal behavior.

There is a large and growing literature that demonstrates that positive connections between youth and parents, peers, and school are protective against suicidal behavior [99]. Therefore, in both assessment and treatment, it is important to ascertain what interpersonal resources are available to the suicidal patient, and what can be done to strength them.

The finding of a blunted HPA response to social stress is a consistent finding across several studies. While the above-cited studies do not address this issue, it is well known that a history of maltreatment can result in the downregulation of the glucocorticoid receptor [100], resulting in a blunted response to social stress. As noted previously, social stressors were more likely to precipitate a suicide attempt in adolescent girls who showed a blunted cortisol reponse to social stress [70].

While there are studies that have not supported the Interpersonal theory of Suicide or the Psychache theory, a recent, large longitudinal study of high-risk adolescent and young adults found that all of the constructs of both of these theories (Perceived Burdensomeness, Thwarted Belonging, Acquired capability [in the study related to fearlessness about death], and psychological pain) all predicted future suicidal behavior, as did a lower level of reasons for living [87]. These provide useful treatment targets for suicidal patients.

There is a paucity of psychological autopsy studies that have compared suicidal ideators to those who die by suicide. A study of young adult soldiers who died by suicide compared them to living soldiers who had experiences SI in the previous 12 months. The cases and controls were quite similar, except that suicide decedents were more likely than suicidal controls to have had 3 or more mental disorders that were present within the past 30 days. In adolescents, the presence of a mood disorder combined with non-affective comorbidity was much more common in suicide decedents than in the control participants. Large epidemiological studies, mostly conducted in adults, have shown that the combination of a mood disorders with either disorders of disinhibition (e.g., substance e abuse, externalizing disorders) or distress (e.g., PTSD) were more closely with suicide attempts than was mood disorder alone [7]. Although there have not been many longitudinal studies of suicidal ideators as a function of sleep quality, results from both longitudinal studies from the Army STARRS study, and from psychological autopsy studies show that sleep difficulties are imminent predictors of suicidal risk. In one study of adolescent suicide, insomnia was five times more common in suicidal decedents than in controls, even after controlling for the severity of depression. Recent meta-analysis and systemic review confirms the role of poor sleep in SI and SA in youth [95,96]. There is evidence that treatment of sleep problems can improve suicidal ideation [96,101].

Clinical Implications: The most impactful risk factors for transition from ideation to attempt are characteristics of the ideation and past suicidal behavior itself: evidence of planning and/or intent, duration, intrusiveness, cause of distress, NSSI, and past suicidal behavior. Any form of psychopathology can increase the likelihood of an attempt, but most prominent of mood disorder comorbid with either disorders of disinhibition (e.g., externalizing disorder, alcohol/substance abuse) or distress (e.g., panic disorder, PTSD)., low distress tolerance, and poor emotional regulation.

Limitations: This review has several limitations that should be considered when interpreting its findings. The heterogeneity in study designs, sample characteristics, and measurement approaches across included studies precluded a formal meta-analysis, requiring a narrative synthesis that limits the precision with which effect sizes can be compared. Methodological quality was variable, with NOS scores ranging from 2 to 8, and several studies relied on retrospective designs or clinical samples that may limit causal inference and generalizability to community populations. It is also worth noting that several studies categorized here as case-control were not originally designed as such, but were analyzed in a case-control framework; while this approach is informative, it may introduce subtle selection and ascertainment biases that would not be present in studies prospectively designed with matched controls. Most studies relied on self-report, which is subject to recall bias and underreporting, a particular concern given that a substantial proportion of suicidal individuals do not disclose their ideation even when directly assessed. Future research should prioritize diverse samples and standardized measurements to address these gaps.

Further research. Studies in adults using EMA have been effective in identifying the factors that affect fluctuations in suicidal ideation and the transition to suicidal behavior; such studies are also needed in adults. Studies using mobile sensing to assess geolocation, activity levels, circadian rhythms, word choice and sentiment have the potential to shed light on imminent risk for suicide. Although safety planning has become *de rigeur* for child and adolescent suicidal patients, the efficacy of this intervention has not been shown to be effective in teens, nor has there been studies about the relationship between the quality of safety planning and its efficacy. Treatment trials that focus on patients with suicidal ideation that target critical variables associated with the transition for SI to SB could help identify the most salient targets to treat to prevent the progression to SB.

## Author contributions

DB conceptualized and designed the study, as well as conducted the initial review. DD developed the search strategy and conducted the literature search, with assistance and supervision from DB. DD screened titles/abstracts and full-text articles for inclusion. DD extracted data and DD and DB stratified the findings into groups of risk factors. DD assessed risk of bias, with DB verifying a subset for accuracy. DB finalized the interpretations of the findings. DB wrote the first draft of the introduction, results, and discussion, while DD wrote the first draft of the methods and abstract section, and created figures and tables. DB and DD critically revised the manuscript. All authors had full access to all data in the study and had final responsibility for the decision to submit for publication. DD and DB had direct access to, and verified, all data reported in the manuscript.

## Data sharing

Study materials such as raw data collection forms, raw scoring forms, and full data used for all analyses, are available upon reasonable request.

## Funding

DD did not receive any funding for this paper. DB was supported in part by 10.13039/100000025NIMH grant P50 MH115838. The funding agency did not review or approve this paper.

## Declaration of competing interest

The authors declare the following financial interests/personal relationships which may be considered as potential competing interests: David Brent reports financial support and administrative support were provided by ETUDES Center. David Brent has patent Guilford Press, The electronic self-rated version of the C-SSRS from eRT, Inc. with royalties paid to David Brent. David Brent has patent Computerized Adaptive Screen for Suicidal Youth (CASSY) issued to David Brent. David Brent has patent BRITE App issued to David Brent. David Brent has patent Screening Wizard issued to David Brent. Dr. Brent receives research support from NIMH, AFSP, the Once Upon a Time Foundation, and the Beckwith Foundation. Also performing duties for UptoDate Inc. Psychiatry Section Editor, and receives consulting fees from Healthwise. If there are other authors, they declare that they have no known competing financial interests or personal relationships that could have appeared to influence the work reported in this paper.
